# Association of dysglycaemia with persistent infarct core iron in patients with acute ST-segment elevation myocardial infarction

**DOI:** 10.1016/j.jocmr.2024.100996

**Published:** 2024-01-17

**Authors:** Ivan Lechner, Martin Reindl, Fritz Oberhollenzer, Christina Tiller, Magdalena Holzknecht, Priscilla Fink, Thomas Kremser, Paolo Bonatti, Felix Troger, Benjamin Henninger, Agnes Mayr, Axel Bauer, Bernhard Metzler, Sebastian J. Reinstadler

**Affiliations:** a University Clinic of Internal Medicine III, Cardiology and Angiology, Medical University of Innsbruck, Anichstrasse 35, A-6020 Innsbruck, Austria; b University Clinic of Radiology, Medical University of Innsbruck, Anichstrasse 35, A-6020 Innsbruck, Austria

**Keywords:** ST-elevation myocardial infarction, HbA1c, Intramyocardial hemorrhage, Persistent iron, Iron resolution, Cardiac magnetic resonance imaging

## Abstract

**Background:**

Dysglycaemia increases the risk of myocardial infarction and subsequent recurrent cardiovascular events. However, the role of dysglycaemia in ischemia/reperfusion injury with development of irreversible myocardial tissue alterations remains poorly understood.

In this study we aimed to investigate the association of ongoing dysglycaemia with persistence of infarct core iron and their longitudinal changes over time in patients undergoing primary percutaneous coronary intervention (PCI) for acute ST-segment elevation myocardial infarction (STEMI).

**Methods:**

We analyzed 348 STEMI patients treated with primary PCI between 2016 and 2021 that were included in the prospective MARINA-STEMI study (NCT04113356). Peripheral venous blood samples for glucose and glycated hemoglobin (HbA1c) measurements were drawn on admission and 4 months after STEMI. Cardiac magnetic resonance (CMR) imaging including T2 * mapping for infarct core iron assessment was performed at both time points. Associations of dysglycaemia with persistent infarct core iron and iron resolution at 4 months were calculated using multivariable regression analysis.

**Results:**

Intramyocardial hemorrhage was observed in 147 (42%) patients at baseline. Of these, 89 (61%) had persistent infarct core iron 4 months after infarction with increasing rates across HbA1c levels (<5.7%: 33%, ≥5.7: 79%). Persistent infarct core iron was independently associated with ongoing dysglycaemia defined by HbA1c at 4 months (OR: 7.87 [95% CI: 2.60–23.78]; p < 0.001), after adjustment for patient characteristics and CMR parameters. The independent association was present even after exclusion of patients with diabetes (pre- and newly diagnosed, n = 16).

**Conclusions:**

In STEMI patients treated with primary PCI, ongoing dysglycaemia defined by HbA1c is independently associated with persistent infarct core iron and a lower likelihood of iron resolution. These findings suggest a potential association between ongoing dysglycaemia and persistent infarct core iron, which warrants further investigation for therapeutic implications.

## Graphical Abstract

**Figure:**
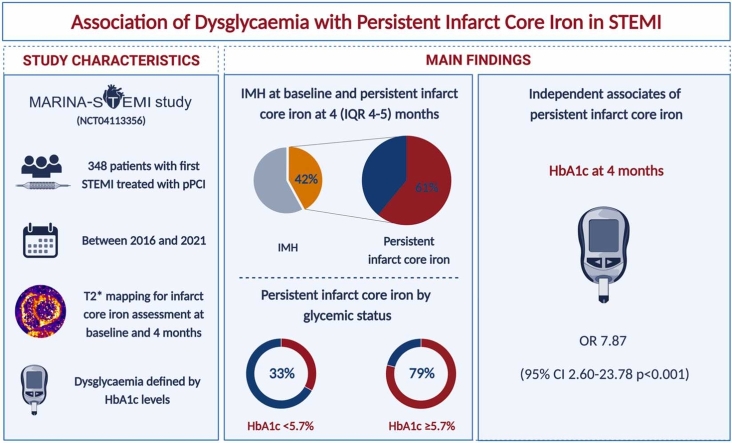

## Background

Incomplete myocardial tissue reperfusion as a consequence of microvascular injury occurs in every second to third patient with acute ST-segment elevation myocardial infarction (STEMI) [1], [2]. The presence and severity of microvascular injury is strongly associated with worse outcomes [3], [4]. Microvascular injury is a complex and heterogeneous phenomenon, mainly caused by severe ischemia/reperfusion injury together with distal embolization of thrombus and individual susceptibility [4]. Cardiovascular magnetic resonance (CMR) imaging has become the reference standard for characterizing microvascular injury in myocardial infarction (MI) patients [4], [5], [6]. Microvascular obstruction (MVO) and intramyocardial hemorrhage (IMH) are the two distinct entities of microvascular injury that can be visualized by CMR with high accuracy [6], [7]. IMH, the most severe form of microvascular injury, is characterized by extravasation of erythrocytes, resulting in iron deposition inside the infarcted myocardium [6]. IMH has recently moved into clinical focus by demonstrating particularly strong prognostic implications in STEMI patients, incremental to other infarct severity parameters including infarct size and MVO [8], [9], [10]. Furthermore, it was recently shown that IMH might not only be a consequence, but rather a determinant of final infarct size [11].

In contrast to MVO, which resolves within the first weeks after STEMI, persistence of infarct core hemorrhage can be detected in the chronic stage after MI in a significant proportion of patients [12] and has been shown to drive a persistent, proinflammatory burden within the infarct zone, leading to left ventricular dysfunction, adverse remodeling, and worse clinical outcomes [1], [2], [8], [10]. The underlying pathophysiology is still not completely understood, however, recent evidence demonstrated that iron-induced macrophage activation drives fatty infiltration of the myocardium contributing to unfavorable cardiac remodeling [13].

Dysglycaemia not only represents a major risk factor for the development of a first-time MI, but is also associated with higher rates of recurrent cardiovascular complications thereafter [14]. Furthermore, it is well known that patients with STEMI and concomitant dysglycaemia have worse short- and long-term clinical outcomes [8], [10]. Considering the adverse effects of dysglycaemia on the microvasculature, and recently published data, demonstrating an association between dysglycaemia and more severe microvascular injury in the acute setting after MI [15], incomplete myocardial reperfusion due to IMH could be one explanation for the adverse outcome in STEMI patients with dysglycaemia [16], [17].

We hypothesized that dysglycaemia in patients suffering acute STEMI might be associated with persistent infarct core iron as depicted by CMR imaging. Therefore, we investigated the association of dysglycaemia with persistence of infarct core iron and their longitudinal changes over time.

## Methods

### Study design

The current investigation is based on the Magnetic Resonance Imaging In Acute ST-Elevation Myocardial Infarction (MARINA-STEMI, NCT04113356) study, a prospective observational study that analyzed 348 STEMI patients at the coronary care unit of Innsbruck Medical University Hospital between 2016 and 2021. Briefly, MARINA STEMI was designed to evaluate the nature and clinical significance of myocardial tissue characteristics as determined by CMR imaging in STEMI patients.

Inclusion criteria were first STEMI, defined by clinical symptoms suggestive of ischemia and significant ST-segment elevation in at least 2 contiguous leads (>0.1 mV in extremity leads; >0.2 mV in precordial leads), who were treated by primary percutaneous coronary intervention (PCI) within 24 h following symptom onset. Exclusion criteria were defined as follows: age < 18 years, an estimated glomerular filtration rate < 30 ml/min/1.73 m², Killip class ≥ 3 at the time of CMR imaging, any history of a previous MI or coronary intervention and any CMR contraindication (pacemaker, orbital foreign body, cerebral aneurysm clip, manifest claustrophobia, known or suggested contrast agent allergy to gadolinium).

The study was conducted in accordance with the Declaration of Helsinki and received approval by the research ethics committee of the Medical University of Innsbruck. Written informed consent was given by all patients before study inclusion.

### Biomarker measurements

Blood samples for glucose and glycated hemoglobin A1c (HbA1c) analyses were obtained via peripheral venipuncture at hospital admission and at 4 months follow-up.

HbA1c at 4 months was available in 245 patients (70% of total cohort).

High-performance liquid chromatography was used for HbA1c measurements following the International Federation for Clinical Chemistry (IFCC) reference method [18]. HbA1c was expressed as percentage according to the National Glycohemoglobin Standardization Program (NGSP) by applying the following IFCC/NGSP ‘master equation’ [19]:

HbA1c (NGSP)% = HbA1c (IFCC) mmol/mol / 10·929 + 2·15

Non-fasting glucose levels were analyzed using whole-blood amperometry. Dysglycaemia was defined as having either pre- or manifest diabetes according to HbA1c values. Prediabetes was defined by a HbA1c value of ≥ 5.7%, manifest diabetes by a HbA1c value of ≥ 6.5% [20].

### Cardiovascular magnetic resonance

CMR imaging was performed on a 1.5 Tesla Magnetom AVANTO-scanner (Siemens®, Erlangen, Germany) within the first week after PCI and 4 months thereafter. Image acquisition and post-processing were performed following a previously published protocol [21]. In brief, short-axis cine images acquired by electrocardiogram (ECG)-triggered balanced steady-state free precession bright-blood sequences were used to evaluate left ventricular volumes and function. Late gadolinium-enhanced (LGE) images were obtained 10–15 min after application of 0.2 mmol/kg contrast medium (Gadovist®, Bayer®, Leverkusen, Germany), applying an ECG-triggered phase-sensitive inversion recovery sequence. Infarct area was defined by hyper-enhancement at a threshold of + 5 standard deviations (SD) above the signal intensity of remote myocardial tissues of the opposite left ventricle. MVO was defined by hypo-enhancement within the infarct area on LGE images.

IMH and persistent infarct core iron were assessed by T2 * mapping using a breath-hold, cardiac gated gradient echo sequence with eight echoes obtained in three matching short-axis slices before administration of the contrast agent. A motion correction algorithm was applied to reduce movement artefacts. Typical imaging parameters were: echo time = 2.02–16.0 ms (ΔTE = 2 ms), time to repetition = 18.2 ms, flip angle = 20, bandwidth = 815 Hz/pixel, In-plane pixel spacing 1.6 × 1.6 mm, and slice thickness = 8 mm. Motion corrected, color-coded T2 * -maps were automatically inline generated by fitting signal intensities at each image pixel with an exponential model for the given echo times.

IMH was defined as a region of hypo-intense core within the infarcted area with a T2 * reduction below 20 ms at baseline. Persistent infarct core iron was defined as a region of hypo-intense core within the infarcted area with T2 * reduction below 20 ms at 4 months follow-up [8]. A region of interest (ROI) size of at least 20 pixels was applied to determine T2 * values.

Inter- and intraobserver variability has been reported previously by our research group [12].

### Statistical analyses

Statistical analysis was performed with SPSS Statistics 29.0.1 (IBM, Armonk, New York), MedCalc Version 22.0.22 (Ostend, Belgium) and R 4.2.0 (The R Foundation, Vienna, Austria). Distribution of data was tested using the Shapiro-Wilk test. Categorical variables are depicted as frequencies with corresponding percentages. Continuous variables are presented as mean ± SD or median with interquartile range (IQR), according to their distribution. Differences between groups were tested with Student’s t-test, Mann–Whitney U-test, or Chi-square test as indicated. Univariable and multivariable logistic regression analyses were conducted to disclose significant and independent associations between persistent infarct core iron and iron resolution (dependent binary variable). All clinical, CMR and angiographic variables, as well as biomarkers of myocardial wall-stress, infarct size, and inflammation at baseline (Table 1) showing a p-value of < 0.10 in univariable testing were entered in the corresponding multivariable regression analysis, using the forced entry method. To allow for a better comparison of odds ratios (OR), all OR are presented for 1 standard deviation increase, unless otherwise stated.

**Table 1:** Baseline characteristics of the study cohort (n = 348).

	Total population (n = 348)	No IMH (n = 201, 58%)	IMH (n = 147, 42%)	p-value
**Patient characteristics**				
Age, years	56 [51–65]	56 [50–65]	57 [52–65]	0.20
Female, n (%)	69 (20)	47 (23)	22 (15)	0.05
Hypertension, n (%)	149 (43)	85 (42)	64 (44)	0.82
Current smoker, n (%)	193 (56)	109 (54)	84 (57)	0.59
Hyperlipidemia, n (%)	183 (53)	113 (56)	70 (48)	0.11
Diabetes mellitus, n (%)	32 (9)	16 (8)	16 (11)	0.35
Admission Glucose, mg/dl	131 [115–154]	130 [114–151]	133 [117–161]	0.10
HbA1c, baseline %	5.6 [5.4-5.9]	5.6 [5.4-5.9]	5.8 [5.5-6.0]	**< 0.001**
HbA1c, 4 months %	5.9 [5.6-6.1]	5.8 [5.6-6.1]	6.0 [5.7-6.1]	**0.07**
Admission Creatinine, mg/dl	0.95 [0.83-1.06]	0.94 [0.84-1.06]	0.95 [0.83-1.10]	0.44
Total ischemic time, min	168 [112-297]	159 [109-270]	195 [119-322]	0.07
Culprit lesion, n (%)				**0.02**
RCA	133 (38)	87 (43)	46 (31)	
LAD	160 (46)	91 (45)	69 (47)	
LCX	53 (15)	23 (12)	30 (21)	
RI	2 (1)	0 (0)	2 (1)	
Number of diseased vessels, n (%)				0.57
1	216 [62]	128 [64]	88 [60]	
2	94 (27)	54 (27)	40 (27)	
3	38 (11)	19 (9)	19 (13)	
Pre-interventional TIMI flow, n (%)				**0.01**
0	223 [64]	114 [57]	108 [73]	
1	51 (15)	30 (15)	21 (14)	
2	46 (13)	32 (16)	14 (10)	
3	28 (8)	24 (12)	4 (3)	
Post-interventional TIMI flow, n (%)				**0.04**
0	2 (0.5)	1 (0.5)	1 (1)	
1	4 (0.5)	1 (0.5)	3 (2)	
2	26 (8)	9 (5)	17 (11)	
3	316 [91]	189 [94]	126 [86]	
Peak hs-cTnT, ng/l	4746 [1997-8233]	3117 [1323-5741]	7291 [4396-13414]	**< 0.001**
NT-pro-BNP, ng/l	1386 [698-2453]	1111 [625-1965]	1897 [1047-3235]	**< 0.001**
Peak hs-CRP, mg/l	25.4 [13.1-46.7]	20.5 [10.3-37.7]	34.3 [17.8-65.3]	**< 0.001**
**Baseline CMR**				
IS, % of LVMM	15 [8–25]	12 [4–18]	24 [14–32]	**< 0.001**
MVO, n (%)	195 [56]	48 (24)	147 (100)	**< 0.001**
MVO, % of LVMM	0.3 [0.0-2.2]	0.0 [0.0-0.4]	2.2 [0.6-5.1]	**< 0.001**
LVEF, %	48 [ ± 9]	51 [ ± 8]	45 [ ± 9]	**< 0.001**
LVEDV, ml	164 [138–189]	161 [134–187]	173 [141–203]	**0.01**
LVESV, ml	84 [64–105]	75 [61–98]	92 [76–119]	**< 0.001**
**4-months CMR**				
IS, % of LVMM	11 [4–18]	8 [2–13]	15 [10–22]	**< 0.001**
Persistent infarct core iron, n (%)	89 26)	0 (0)	89 [61]	**< 0.001**
LVEF, %	52 [44–58]	55 [49–60]	46 [39–54]	**< 0.001**
LVEDV, ml	163 [135–192]	157 [128–183]	174 [144–207]	**< 0.001**
LVESV, ml	78 [59–106]	69 [54–90]	94 [73–118]	**< 0.001**

Abbreviations: IMH=Intramyocardial hemorrhage, HbA1c=Glycated hemoglobin, RCA=Right coronary artery, LAD=Left anterior descending artery, LCX=Left circumflex artery, RI=Ramus intermedius, TIMI=Thrombolysis in myocardial infarction, hs-cTnT=high-sensitivity cardiac Troponin T, NT-pro-BNP=N-terminal pro-B-type natriuretic peptide, hs-CRP=high-sensitivity C-reactive protein, CMR=Cardiac magnetic resonance, IS=Infarct size, LVMM=Left ventricular myocardial mass, MVO=Microvascular obstruction, LVEF=Left ventricular ejection fraction, LVEDV=Left ventricular end-diastolic volume, LVESV=Left ventricular end-systolic volume, ml=milliliters, ¶=presented as mean ± standard deviation.

The multivariable logistic regression model was also subjected to sensitivity analysis to estimate relative risks. Risk ratios were calculated using the generalized linear model with binomial family using R 4.2.0. For sensitivity analysis, HbA1c at 4 months was categorized according to clinically established cut-offs (HbA1c <5.7%, HbA1c 5.7–6.4, HbA1c ≥6.5%). Other continuous variables were dichotomized according to their median.

Major adverse cardiovascular events (MACE), defined as all-cause death, myocardial reinfarction, and new congestive heart failure, were assessed during 4-months follow-up using a standardized questionnaire. All reported endpoints were checked afterwards by carefully reviewing the medical records. In patients with more than one event during follow-up, the most severe endpoint was used for the composite endpoint (all-cause death > myocardial reinfarction > new congestive heart failure) [22].

A two-tailed p-value of < 0.05 was considered as statistically significant.

## Results

### Baseline characteristics and dysglycaemia

Baseline characteristics of the study cohort according to the presence (42%) and absence (58%) of IMH at baseline are shown in Table 1. Median HbA1c at admission was 5.6 (interquartile range [IQR]: 5.4–5.9) %, and median admission glucose concentration was 131 (IQR: 115–154) mg/dl. Median HbA1c at 4 months was 5.9 (IQR: 5.6–6.1) %. Change of HbA1c from baseline to 4 months was + 0.3%. Of the overall cohort, 32 patients (9%) had previously known diabetes. Patients with pre-diagnosed diabetes had significantly higher HbA1c levels (7.6 [IQR: 6.6–8.7] vs. 5.6 [IQR: 5.4–5.9] %; p < 0.01) as well as glucose concentrations (208 [IQR: 175–272] vs. 128 [IQR: 114–146] mg/dl; p < 0.01) than patients without previously known diabetes. Among patients without previously known diabetes (n = 316), the HbA1c value classified 178 patients (56%) as non-diabetics, 128 patients (41%) as having prediabetes, and 10 patients (3%) as newly diagnosed diabetics. Concomitant medication at 4 months is provided in the supplementary section.

### Dysglycaemia and infarct characteristics

CMR parameters at baseline and 4 months according to the presence and absence of IMH are shown in detail in Table 1.

CMR scans were performed 4 (IQR: 3–5) days and 4 (IQR: 4–5) months after PCI.

In total, 195 (56%) patients showed MVO, median MVO size was 0.3 (IQR: 0.0–2.2) % of left ventricular myocardial mass (LVMM). Baseline IMH was present in 147 (42%) patients. Patients with IMH presented with significantly higher baseline HbA1c values as compared to those without IMH (5.8 [IQR: 5.5–6.0] vs. 5.6 [IQR: 5.4–5.9] %; p < 0.001), whereas HbA1c values at 4 months did not significantly differ between groups (6.0 [IQR: 5.7–6.1] vs. 5.8 [IQR: 5.6–6.1] %; p = 0.07).

**Fig. 1:**
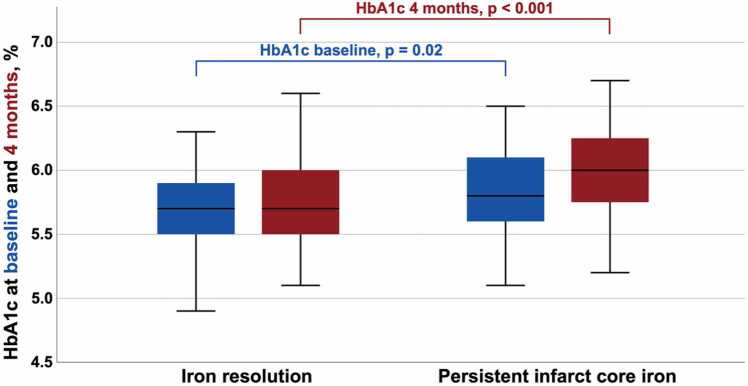
Association of HbA1c and persistent infarct core iron and iron resolution in patients with IMH. Abbreviations: HbA1c=Glycated hemoglobin, IMH= Intramyocardial hemorrhage.

Of the 147 patients with IMH at baseline, 89 (61%) had persistent infarct core iron, with increasing rates across HbA1c levels (<5.7%: 33%, ≥5.7: 79%). Resolution of infarct core iron from baseline to 4 months occurred in 58 (39%) patients. Patients with persistent infarct core iron presented with significantly higher values of HbA1c at baseline and 4 months, as compared to patients with iron resolution (5.8 [IQR: 5.6–6.1] vs. 5.7 [IQR: 5.5–5.9]%; p = 0.02 and 6.0 [IQR: 5.7–6.3] vs. 5.7 [IQR: 5.5–6.0]%; p < 0.001, respectively) (Table 2, Fig. 1). Furthermore, patients with persistent infarct core iron had more commonly hyperlipidemia (55 vs. 36%; p = 0.03) and presented with higher LVESV (175 [IQR: 145–214] vs. 168 [IQR: 136–186] ml, p = 0.01) at baseline and lower left ventricular ejection fraction (LVEF) at 4 months (45 [IQR: 36–51] vs. 48 [IQR: 41–57]%; p = 0.02), respectively.

**Table 2:** Baseline characteristics of patients with intramyocardial hemorrhage (n = 147).

	No persistent iron (n = 58, 39%)	Persistent iron (n = 89, 61%)	p-value
**Patient characteristics**			
Age, years	57 [52–67]	57 [52–64]	0.52
Female, n (%)	9 (16)	13 (15)	0.88
Hypertension, n (%)	29 (50)	35 (39)	0.20
Current smoker, n (%)	32 (55)	52 (58)	0.70
Hyperlipidemia, n (%)	21 (36)	49 (55)	**0.03**
Diabetes mellitus, n (%)	5 (9)	11 (12)	0.48
Admission Glucose, mg/dl	127 [116–151]	137 [120–164]	0.24
HbA1c, baseline %	5.7 [5.5-5.9]	5.8 [5.6-6.1]	**0.02**
HbA1c, 4 months %	5.7 [5.5-6.0]	6.0 [5.7-6.3]	**< 0.001**
Admission Creatinine, mg/dl	0.97 [0.86-1.13]	0.93 [0.81-1.09]	0.72
Total ischemic time, min	224 [128-326]	184 [117-322]	0.26
Culprit lesion, n (%)			0.20
RCA	15 (26)	31 (35)	
LAD	27 (47)	42	
LCX	14 (24)	16 (18)	
RI	2 (3)	0 (0)	
Number of diseased vessels, n (%)			0.36
1	38 (65)	50 (56)	
2	12 (21)	28 (32)	
3	8 (14)	11 (12)	
Pre-interventional TIMI flow, n (%)			0.93
0	41 (71)	67 (75)	
1	9 (16)	12 (14)	
2	6 (10)	8 (9)	
3	2 (3)	2 (2)	
Post-interventional TIMI flow, n (%)			0.15
0	0 (0)	1 (1)	
1	2 (4)	1 (1)	
2	3 (5)	14 (16)	
3	53 (91)	73 (82)	
Peak hs-cTnT, ng/l	6683 [3952-13500]	7511 [4827-13506]	0.34
NT-pro-BNP, ng/l	1793 [1103-3165]	1940 [1018-3297]	0.83
Peak hs-CRP, mg/l	32 [17.8-55.5]	36.7 [18.0-72.5]	0.32
**Baseline CMR**			
IS, % of LVMM^¶^	23 [± 11]	24 [± 12]	0.44
MVO, n (%)	58 (100)	89 (100)	-
MVO, % of LVMM	1.6 [0.8-3.8]	3.0 [0.4-6.4]	0.15
LVEF, %^¶^	46 [± 10]	43 [± 9]	0.07
LVEDV, ml	168 [136–186]	175 [145–214]	0.05
LVESV, ml	168 [136–186]	175 [145–214]	**0.01**
**4-months CMR**			
IS, % of LVMM	13 [9–21]	17 [10–22]	0.19
LVEF, %	48 [41–57]	45 [36–51]	**0.02**
LVEDV, ml	171 [± 41]	179 [± 52]	0.37
LVESV, ml	87 [70–113]	97 [75–129]	0.09

Abbreviations: IMH=Intramyocardial hemorrhage, HbA1c=Glycated hemoglobin, RCA=Right coronary artery, LAD=Left anterior descending artery, LCX=Left circumflex artery, RI=Ramus intermedius, TIMI=Thrombolysis in myocardial infarction, hs-cTnT=high-sensitivity cardiac Troponin T, NT-pro-BNP=N-terminal pro-B-type natriuretic peptide, hs-CRP=high-sensitivity C-reactive protein, CMR=Cardiac magnetic resonance, IS=Infarct size, LVMM=Left ventricular myocardial mass, MVO=Microvascular obstruction, LVEF=Left ventricular ejection fraction, LVEDV=Left ventricular end-diastolic volume, LVESV=Left ventricular end-systolic volume, ml=milliliters, ¶=presented as mean ± standard deviation.

**Fig. 2:**
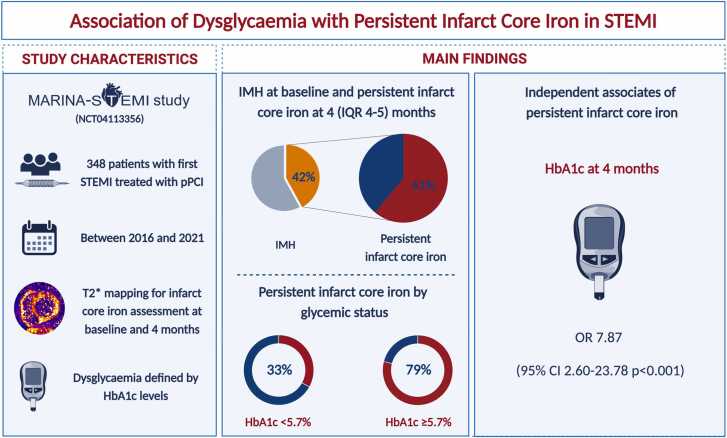
Key Illustration. *Created with BioRender.com, OR are presented per 1 SD increase*. Abbreviations: STEMI=ST-elevation myocardial infarction, pPCI=Primary percutaneous intervention, HbA1c=Glycated hemoglobin, IQR=Interquartile range, OR=Odds ratio.

In univariable regression analysis, HbA1c at 4 months was significantly associated with persistent infarct core iron (OR: 5.14 [95% CI: 2.07–12.76]; p < 0.001). Baseline HbA1c was not significantly associated with persistent infarct core iron (p > 0.05). After adjustment for univariable associates (patient characteristics, biomarkers and CMR parameters), of these variables, only HbA1c at 4 months was independently associated with persistent infarct core iron in multivariable regression analysis (OR: 7.87 [95% CI: 2.60–23.78]; p < 0.001, Fig. 2).

The independent association between HbA1c at 4 months and persistent infarct core iron was significant irrespective of whether HbA1c was modeled as continuous (Table 3), or dichotomized (≥5.7%) variable (OR: 3.38 [95% CI: 1.88–6.05]; p < 0.001).

**Table 3:** Logistic regression analysis for the prediction of persistent infarct core iron.

	Univariable analysis		Multivariable analysis	
	OR (95%CI)	*P*value	OR (95%CI)	*P*value
Age	1.01 (0.73-1.40)	0.96	-	-
Female sex	0.97 (0.67-1.41)	0.88	-	-
Hypertension	0.81 (0.58-1.12)	0.20	-	-
Current smoker	1.07 (0.77-1.49)	0.70	-	-
Hyperlipidemia	1.47 (1.05-2.06)	**0.03**	1.26 (0.76-2.09)	0.36
Diabetes mellitus	1.12 (0.81-1.55)	0.48	-	-
Admission Glucose	1.25 (0.88-1.77)	0.22	-	-
HbA1c, baseline	1.34 (0.90-2.00)	0.15	-	-
HbA1c, 4 months	5.14 (2.07-12.76)	**< 0.001**	7.87 (2.60-23.78)	< 0.001
Admission Creatinine	1.06 (0.80-1.40)	0.70	-	-
Total ischemic time	0.77 (0.56-1.06)	0.10	-	-
Culprit lesion	0.74 (0.54-1.02)	**0.06**	0.83 (0.49-1.39)	0.47
Number of diseased vessels	1.11 (0.81-1.54)	0.51	-	-
Pre-interventional TIMI flow	0.87 (0.57-1.33)	0.52	-	-
Post-interventional TIMI flow	0.83 (0.61-1.14)	0.26	-	-
Peak hs-cTnT	1.19 (0.87-1.63)	0.29	-	-
NT-pro-BNP	1.08 (0.81-1.43)	0.60	-	-
Peak hs-CRP	1.19 (0.87-1.62)	0.27	-	-
IS, % of LVMM	1.15 (0.81-1.62)	0.44	-	-
MVO, % of LVMM	1.46 (1.05-2.04)	**0.02**	1.09 (0.63-1.89)	0.76
LVEF	0.73 (0.52-1.03)	**0.07**	1.08 (0.10-12.22)	0.95
LVEDV	1.48 (1.05-2.09)	**0.03**	0.54 (0.01-22.24)	0.75
LVESV	1.60 (1.12-2.28)	**0.01**	4.65 (0.03-633.92)	0.54

Abbreviations: OR=Odds ratio, CI=confidence interval, HbA1c=Glycated hemoglobin, TIMI=Thrombolysis in Myocardial Infarction, hs-cTnT=High sensitivity cardiac troponin-T,NT-pro-BNP=N-terminal pro-B-type natriuretic peptide, hs-CRP=High sensitivity c-reactive protein, IS=Infarct size, LVMM=Left ventricular myocardial mass, MVO=Microvascular obstruction, LVEF=Left ventricular ejection fraction, LVEDV= Left ventricular end-diastolic volume, LVESV=Left ventricular end-systolic volume. OR are presented per 1 SD increase.

In a further model, where HbA1c at 4 months was categorized into clinically relevant cut-offs (HbA1c <5.7%, 5.7–6.4, ≥6.5%), the results remained consistent with our original findings, demonstrating an independently association of HbA1c groups and persistent infarct core iron (OR: 8.27 [95% CI: 2.73–25.02], p < 0.001) (Table S1).

Sensitivity analyses further confirmed these findings, showing a risk ratio for HbA1c categories of 7.69 (95% CI: 2.74–21.56, p < 0.001), after adjustment for univariable associates (median left ventricular end-systolic volume at baseline, median MVO size, hyperlipidemia).

The exclusion of patients with pre-diagnosed (n = 16) and newly diagnosed (HbA1c ≥6.5%, n = 5) diabetes resulted in a cohort of 126 patients. In this “non-diabetic” cohort, 72 patients (57%) had pre-diabetes (defined by HbA1c ≥5.7%). In these patients a numerically higher rate of persistent infarct core iron (65 vs. 52%), as compared to patients with HbA1c < 5.7% was observed. HbA1c at 4 months remained significantly related to persistent infarct core iron in multivariable analysis after adjustment for univariable associates (hyperlipidemia, total ischemic time, culprit lesion, MVO size, LVEF, left ventricular end-diastolic volume (LVEDV) and left ventricular end-systolic volume (LVESV) at baseline): OR: 6.36 [95% CI: 2.06–19.60]; p = 0.01) in this cohort as well.

### Clinical outcome

Follow-up data were available in all patients. During the 4-months follow-up period, 13 (4%) patients experienced a MACE, including, 3 (1%) myocardial reinfarctions and 10 (3%) new congestive heart failures. No deaths were observed during follow-up.

A MACE was significantly more common in patients with persistent infarct core iron as compared to patients without persistent infarct core iron at 4 months follow-up (9 vs. 2%, p = 0.01).

## Discussion

This study is the first to investigate a possible association of dysglycaemia with persistent infarct core iron, determined by T2 * -mapping, in a well-characterized cohort of STEMI patients treated according to current practice. The main finding was that higher HbA1c levels at 4 months follow-up were significantly and independently associated with persistent infarct core iron and a lower likelihood of iron resolution at 4 months after infarction. These findings suggest a pathophysiological link between dysglycaemia, persistence of infarct core iron and worse outcome after STEMI. Whether this pathophysiological link also represents an effective target for intervention should be investigated in further studies.

### Intramyocardial hemorrhage in STEMI

In patients suffering STEMI, IMH and its persistence (most often referred to as “persistent infarct core iron”) are increasingly studied tissue biomarkers that can be assessed by T2 * mapping. The presence of IMH drives delayed infarct healing and is responsible for fatty degeneration of the infarcted myocardium contributing to adverse remodeling and ultimately more frequent adverse clinical events [8], [12], [13]. Previous studies using T2 * mapping reported IMH incidence rates of 23 to 50% in the acute setting after PCI for STEMI [7], [8], [9]. Similarly, the present investigation found IMH in 42% of patients in the early phase after infarction.

In contrast to MVO, which resolves in the first weeks after STEMI, persistent infarct core iron, as a consequence of IMH, can be found in the chronic stage after MI in a substantial subset of patients [12]. In the present study, 61% of patients with IMH at baseline showed persistence of infarct core iron at 4 months after MI, which is in line with previous observations [23]. Interestingly, it has recently been demonstrated that persistent infarct core iron can even remain up to a decade after STEMI and its presence is related to poor infarct healing [12]. Furthermore, persistent infarct core residues were shown to be associated with a 4-fold increase in all-cause death and heart failure [23]. During the 4 months of follow-up in our study, we did not observe any deaths. However, a significant increase in the incidence of MACE was observed among patients with persistent infarct core iron. This increased incidence was primarily driven by a significant increase in new congestive heart failure.

In summary, IMH and persistent infarct core iron, assessed via T2 * mapping, are prevalent biomarkers associated with poor infarct healing and adverse outcome, persisting chronically in a significant subset of STEMI patients.

### Dysglycaemia as a determinant of persistent infarct core iron in STEMI

Due to previous observations describing an association between dysglycaemia and microvascular damage in different organ systems [16], as well as impaired outcome in STEMI [14], [24], a pathophysiological link between dysglycaemia and development of irreversible microvascular tissue injury can be assumed.

Although a number of previous studies evaluated the association between dysglycaemia and IMH in the acute setting after PCI for STEMI [9], [17], [25], [26], data on the association between ongoing dysglycaemia and persistent infarct core iron in the chronic phase after STEMI are completely lacking. Furthermore, in most studies investigating the association between dysglycaemia and infarct characteristics, the definition of dysglycaemia relies on patient-reported diabetes diagnosis, hence the number of undiagnosed diabetes as well as prediabetes remains unclear. HbA1c, however, a measure of average blood glucose concentrations over weeks offers a more objective marker to describe dysglycaemia.

In the current investigation, dysglycaemia, defined by HbA1c levels at 4 months, was significantly and independently associated with persistent infarct core iron at 4 months after MI, even after adjustment for established biomarkers of infarct core iron, such as high-sensitivity cardiac troponin T [10]. This study therefore expands previous data by demonstrating that dysglycaemia during STEMI might be an important and potentially modifiable risk factor for irreversible microvascular tissue injury that persists at least for up to 4 months post-MI. Interestingly, only a minority in our study had manifest diabetes, and therefore the primary finding was predominantly driven by patients with prediabetes. This is further corroborated since the exclusion of patients with manifest diabetes strengthened the association of HbA1c and persistent iron residues. One might therefore speculate that those patients with prediabetes could particularly benefit from interventions targeting dysglycaemia. In fact, IMH evolves progressively after STEMI (peak at day 3 post-infarction) [10] and decreases to some extent during the chronic phase after infarction [10]. These findings could potentially indicate that IMH is susceptible to precise preventive measures or therapeutic strategies, should they be identified and established. Furthermore, it has recently been shown that IMH may not only be an expression of larger infarcts per se, but also a determinant of final infarct size itself. In fact, Liu and colleagues demonstrated that IMH was associated with a 3-fold loss of salvageable myocardium, resulting in an almost 2-fold increase in final infarct size in a canine model [11]. Conclusively, dysglycaemia, primarily driven by prediabetes and identified through HbA1c levels at admission, was found to be a significant, potentially modifiable factor associated with persistent infarct core iron in STEMI patients, suggesting a need for precise preventive measures or therapeutic strategies.

### Infarct core iron resolution

The underlying pathophysiological mechanisms by which dysglycaemia leads to persistence of infarct core iron are not completely understood and were not the scope of the present investigation. However, cell signaling dysfunction, formation of toxic metabolites (esp. advanced glycation end products) and altered redox potential due to preceding dysglycaemia might promote extensive and irreversible myocardial tissue damage, with less influence on reversible changes of the myocardial microvasculature [16], [27]. These hypotheses are strengthened by a large amount of preclinical evidence that dysglycaemia and diabetes can exacerbate myocardial ischemia/reperfusion injury [15], [28].

Interestingly, our analysis revealed that ongoing dysglycaemia (defined by HbA1c levels at 4 months) was significantly and independently associated with impaired iron resolution. It is therefore possible, that novel strategies targeting dysglycaemia beginning with HbA1c levels above 5.7 could mitigate the severity of persisting infarct core iron and improve outcome. It is important to emphasize that this is a hypothesis-generating observation and, as such, calls for rigorous investigation in future studies.

### Clinical implications and potential therapeutic strategies for persistent infarct core iron

Prediabetes was observed in 38% of patients. In view of these findings, one could speculate that this so far neglected group of STEMI patients could benefit from early antidiabetic treatment to positively influence the acute and chronic pathophysiological processes leading to persistent core iron.

Data from previous preclinical and clinical studies indicate a possible benefit of insulin in preventing ischemia/reperfusion injury in the setting of acute MI [29]. The effects of insulin that potentially attenuate ischemia/reperfusion damage were reported to go beyond glucose-lowering, including anti-inflammatory, anti-thrombotic, anti-oxidative and anti-apoptotic cascades [30]. Randomized studies testing the clinical benefit of insulin treatment in this setting are, however, very scarce. Furthermore, the existing literature on insulin treatment and hard clinical endpoints after infarction is conflicting [31], [32]. The possible effects of newer antidiabetic agents (e.g. sodium-glucose cotransporter 2 inhibitors) on infarct-related microvascular injury processes remain speculative and are currently being investigated (NCT04899479).

Our results thus suggest that screening for prediabetes and aiming for an optimal glycemic status post-STEMI may be advisable for these patients. Whether meaningful benefits accrue from specific interventions in STEMI patients with altered glucose metabolism should be further investigated.

### Limitations

The observational design of our study limits the ability to definitively establish a causal relationship between HbA1c and persistent infarct core iron. However, the potential causal role of dysglycaemia in the persistence of infarct core iron is a compelling hypothesis and underscores the need for further investigation. Future experimental studies are needed to definitively establish a direct causal relationship and provide deeper insights into the dynamics of this relationship.

Our findings are not generalizable to unstable STEMI patients and those where a CMR examination was not possible. While our study provides important insights, it is important to emphasize that our reliance on T2 * maps derived from only three short-axis slices does not provide a complete representation of the left ventricle. Consequently, small regions of IMH or persistent infarct core iron may have been missed, and volumetric measurement of the infarct core over the entire ventricle was not possible. Another important limitation of this study is the lack of IMH and persistent infarct core iron quantification. Therefore, the relationship between the degree of dysglycaemia and the size of iron deposition cannot be determined. These limitations could potentially affect the precision of our findings and are an area for methodological improvement in future studies. Furthermore, follow-up HbA1c measurements were available in only 70% of the total cohort which represents another important limitation with respect to the secondary aim. Finally, the use of a single HbA1c measurement may not fully reflect the patient's glycemic status throughout the period from infarction to the 4-month follow-up.

## Conclusion

In STEMI patients treated by contemporary primary PCI, ongoing dysglycaemia defined by HbA1c was significantly and independently associated with persistent infarct core iron and a lower likelihood of iron resolution. These data suggest a potential prognostically relevant pathophysiological relationship between dysglycaemia and irreversible microvascular damage after PCI for STEMI. Further research could explore whether dysglycaemia might serve as a therapeutic target to influence persistent infarct core iron and potential outcomes.

## Ethics approval and consent to participate

Ethical approval for this study was granted by the local research ethics committee (Ethics Committee of the Medical University of Innsbruck, Austria; AN3775 281/4.15 405/5.2; 4480a). Written informed consent was given by all patients before study inclusion.

## Funding

The study was supported by grants from the '10.13039/501100015797Austrian Society of Cardiology', 'Tiroler Wissenschaftsförderung' and the '10.13039/501100002428Austrian Science Fund' (FWF grant KLI 772).

## CRediT authorship contribution statement

**Holzknecht Magdalena:** Data curation, Investigation, Methodology, Writing – review & editing. **Tiller Christina:** Data curation, Investigation, Methodology, Writing – review & editing. **Metzler Bernhard:** Conceptualization, Formal analysis, Funding acquisition, Investigation, Methodology, Project administration, Resources, Supervision, Validation, Writing – review & editing. **Reindl Martin:** Conceptualization, Formal analysis, Funding acquisition, Investigation, Methodology, Validation, Writing – review & editing. **Bauer Axel:** Investigation, Methodology, Project administration, Resources, Software, Supervision, Writing – review & editing. **Lechner Ivan:** Conceptualization, Data curation, Formal analysis, Investigation, Methodology, Project administration, Software, Visualization, Writing – original draft, Writing – review & editing. **Mayr Agnes:** Data curation, Formal analysis, Investigation, Methodology, Project administration, Resources, Software, Supervision, Validation, Writing – review & editing. **Reinstadler Sebastian Johannes:** Conceptualization, Data curation, Formal analysis, Funding acquisition, Investigation, Methodology, Project administration, Software, Supervision, Validation, Visualization, Writing – review & editing. **Henninger Benjamin:** Data curation, Formal analysis, Investigation, Methodology, Resources, Software, Writing – review & editing. **Troger Felix:** Data curation, Formal analysis, Investigation, Methodology, Software, Writing – review & editing. **Bonatti Paolo:** Data curation, Investigation, Methodology, Writing – review & editing. **Kremser Thomas:** Data curation, Investigation, Methodology, Writing – review & editing. **Fink Priscilla:** Data curation, Investigation, Methodology, Writing – review & editing. **Oberhollenzer Fritz:** Data curation, Investigation, Methodology, Writing – review & editing.

## Declaration of Competing Interest

The authors declare that they have no known competing financial interests or personal relationships that could have appeared to influence the work reported in this paper.
