# Supplementary material for: Association of dysglycaemia with persistent infarct core iron in patients with acute ST-segment elevation myocardial infarction
Source: J Cardiovasc Magn Reson. 2024 Jan 17;26(1):100996. doi: 10.1016/j.jocmr.2024.100996 (PMC11211234; doi:10.1016/j.jocmr.2024.100996)
Supplement: Supplementary file 1 — Supplementary material [file mmc1.docx]

**Supplementary Material**

**Pre- and concomitant medication at 4 months.**

In total, 28 patients (8%) received pre-medication with oral antidiabetics. Seven patients (2%) received insulin as part of the antidiabetic treatment regime. Other previous medication was reported as follows: Aspirin in 20 patients (6%), beta-blockers in 25 patients (7%), angiotensin-converting-enzyme inhibitors / angiotensin II receptor type 1 antagonists in 72 patients (21%) and statins in 29 patients (8%).

At 4 months follow-up 24 patients (7%) were treated with oral antidiabetics, 12 patients (7%) were treated with insulin. Other concomitant medication were: Aspirin in 327 patients (94%), P2Y_12_-inhibitors in 338 (97%), beta-blockers 302, (87%), angiotensin-converting-enzyme inhibitors / angiotensin II receptor type 1 antagonists in 294 (85%) patients. Statins were received by 333 (96%).

| Table S1: Logistic regression analysis for the prediction of persistent infarct core iron using clinically established HbA1c categories | | | | | | | | |
| --- | --- | --- | --- | --- | --- | --- | --- | --- |
|  | Univariable analysis | |  | |  | Multivariable analysis | |  |
|  | OR (95%CI) | *P* value | |  | | OR (95%CI) | *P* value | |
| Hyperlipidemia | 2.16 (1.09-4.26) | 0.03 | |  | | - | *n.s.* | |
| HbA1c, 4 months (grouped)^¶^ | 5.88 (2.36-14.62) | <0.001 | |  | | 8.27 (2.73-25.02) | <0.001 | |
| Culprit lesion | 0.67 (0.44-1.02) | 0.06 | |  | | - | *n.s.* | |
| MVO, % of LVMM | 1.12 (1.02-1.24) | 0.02 | |  | | - | *n.s.* | |
| LVEF | 0.97 (0.93-1.00) | 0.07 | |  | | - | *n.s.* | |
| LVEDV | 1.01 (1.00-1.02) | 0.03 | |  | | - | *n.s.* | |
| LVESV | 1.02 (1.00-1.03) | 0.01 | |  | | - | *n.s.* | |

Abbreviations: OR=Odds ratio, CI=confidence interval, HbA1c=Glycated hemoglobin, MVO=Microvascular obstruction, LVMM=Left ventricular myocardial mass, LVEF=Left ventricular ejection fraction, LVEDV= Left ventricular end-diastolic volume, LVESV=Left ventricular end-systolic volume, ¶= HbA1c <5.7%, ≥5.7-6.4, ≥6.5%), OR presented as actual values without standardization for 1 standard deviation increase.
